# Analysis of Long Term Study Indicates Both Agronomic Optimal Plant Density and Increase Maize Yield per Plant Contributed to Yield Gain

**DOI:** 10.1038/s41598-018-23362-x

**Published:** 2018-03-21

**Authors:** Yared Assefa, Paul Carter, Mark Hinds, Gaurav Bhalla, Ryan Schon, Mark Jeschke, Steve Paszkiewicz, Stephen Smith, Ignacio A. Ciampitti

**Affiliations:** 1 Department of Agronomy, Kansas State University, 2004 Throckmorton Plant Science Center, Manhattan, Kansas 66506 USA; 2 DuPont Pioneer, 7100 NW, 62nd Ave., Johnston, IA 50131 USA; 3 Department of Agronomy, Iowa State University, 716 Farm House Lane, Ames, IA 50011 USA

## Abstract

Concurrent to yield, maize (*Zea Mays* L.) plant density has significantly increased over the years. Unlike yield, however, the rate of change in plant density and its contribution to maize yield gain are rarely reported. The main objectives of this study were to examine the trend in the agronomic optimum plant density (AOPD) and quantify the contribution of plant density to yield gain. Maize hybrid by seeding rate trials were conducted from 1987–2016 across North America (187,662 data points). Mixed model, response surface, and simple linear regression analyses were applied on the meta-data. New outcomes from this analysis are: (i) an increase in the AOPD at rate of 700 plant ha^−1^ yr^−1^, (ii) increase in the AOPD of 1386, 580 and 404 plants ha^−1^ yr^−1^ for very high yielding (VHY, > 13 Mg ha^−1^), high yielding (HY, 10–13 Mg ha^−1^) and medium yielding (MY, 7–10 Mg ha^−1^), respectively, with a lack of change for the low yielding (LY, < 7 Mg ha^−1^) environment; (iii) plant density contribution to maize yield gain ranged from 8.5% to 17%, and (iv) yield improvement was partially explained by changes in the AOPD but we also identified positive impacts on yield components as other sources for yield gain.

## Introduction

Average plant density for maize (*Zea mays* L.) has increased over the years in the United States^1^, Canada^2^, Brazil^3^, China^4^, and other corn producing countries^5,6^. In many cases, yield increase for modern hybrids was attributed primarily to increase plant density rather than to increase per-plant yield^7,8^. In fact, researchers recently suggested that per-plant yield potential remains unchanged while performance of maize hybrids in high plant density improves, even if these two traits (per-plant yield and density tolerance) are not anatagonstic^9^. Duvick^1^ stated that yield gain due to plant density is perhaps the only clear and quantifiable change in maize hybrids over time. However, unlike yield, the rate of historical change in plant density and the proportion of yield gain attributed to plant density for North America are not yet documented for modern maize hybrids. A plant density increase of about 19000 plants ha^−1^ for the years 1930–1970 and a 21% contribution of plant density to yield gain were reported for Minnesota^10^. Prior to that, a 16% contribution to yield gain for the 1929–1962 period was attributed to changes in plant density for Iowa^11^. An increasing trend of plant density at rate of 597 plants ha^−1^ yr^−1^ for the years 1939–2009 was reported for Kansas^12^. Recently, a review paper on maize and N use efficiency (NUE)^13^, synthesizing 100 scientific publications, presented an overall plant density change from 5.6 plants m^−2^ for years 1940–1990 to 7.1 plants m^−2^ for the period 1991–2011. Both of the most recent studies reporting changes in maize management practices^12^ and NUE^13^ over time were neither focused on estimating yield gains nor in changes based on the agronomic optimum plant density (AOPD).

Planting at the AOPD is among one of the most critical management decisions for maize production because modern hybrids on average have one productive ear per plant and hardly tiller even with an occasional abundance of resource^14,15^. Maize yield is a function of the four yield components, i.e., number of plants per area (plant density), number of ears per plant, number of grains per ear, and grain weight. In an ideal environment with unlimited resources, the relationship between plant density and yield should be linear, i.e., with yield increasing as plant density increases with a slope that equals to the product of ears per plant, number of grains per ear, and grain weight. However, factors such as nutrients, water, and weather are most commonly limiting production, causing the relationship between plant density and yield to follow a quadratic curve with different agronomic optimum points dependent on magnitude of resource limitation^16^. A supra-optimal plant density level, above the resources available to sustain each plant, promotes competition and results in less than the potential combination of ear per plant x grain number x grain weight, and increases plant barrenness^17,18^.

The impact of plant density on yield is dependent on complex interactions between genotype (G), environment (E), and management (M) factors (G × E × M). In drought conditions, for example, Lobell *et al*.^19^ reported that increasing plant density decreased yield and increased yield variability. Ruffo *et al*.^20^ evaluated five management factors that contributed to decreases in the maize yield gap (attainable minus actual yield) and concluded that plant density increased yield only when other management factors (e.g., transgenic insect resistance, fungicides containing strobilurin, N-P–S–Zn fertility) were jointly applied. Previous studies also suggested that the AOPD varied relative to water supply^21^, soil type^22^, and hybrid^23^. Following this rationale, it is essential to isolate the plant density contribution to yield gain, while acknowledging that changes in plant density itself is part of the complex G × E × M interactions. Thus, any investigation geared to identify the sole contribution of plant density over time needs to consider comparison of events at similar environments or yield changes at similar plant density levels.

Since plant density is an important yield component contributing to yield gain, two scientific knowledge gaps were identified related to: i) historical changes in the rate of plant density, and ii) its contribution to yield increase to foster maize yield improvement. As indicated above, there was limited information on the rate of change in plant density at the AOPD for North America over the past few decades. Yield-density relationships should be evaluated at the AOPD for each historical period to quantify a more realistic maize yield gain, and exploring changes in plant density by yield environment, and by latitudes. Therefore, the main objectives of this synthesis-analysis were to examine the trend in the agronomic optimum plant density (AOPD) and quantify the contribution of plant density to yield gain. Analysis of AOPD trend over time was conducted by latitude and yield environments. We also provided an insight on the changes in the yield-to-plant density association relative to yield components.

## Results

### Trend in agronomic optimum plant density (AOPD)

**Figure 1:**
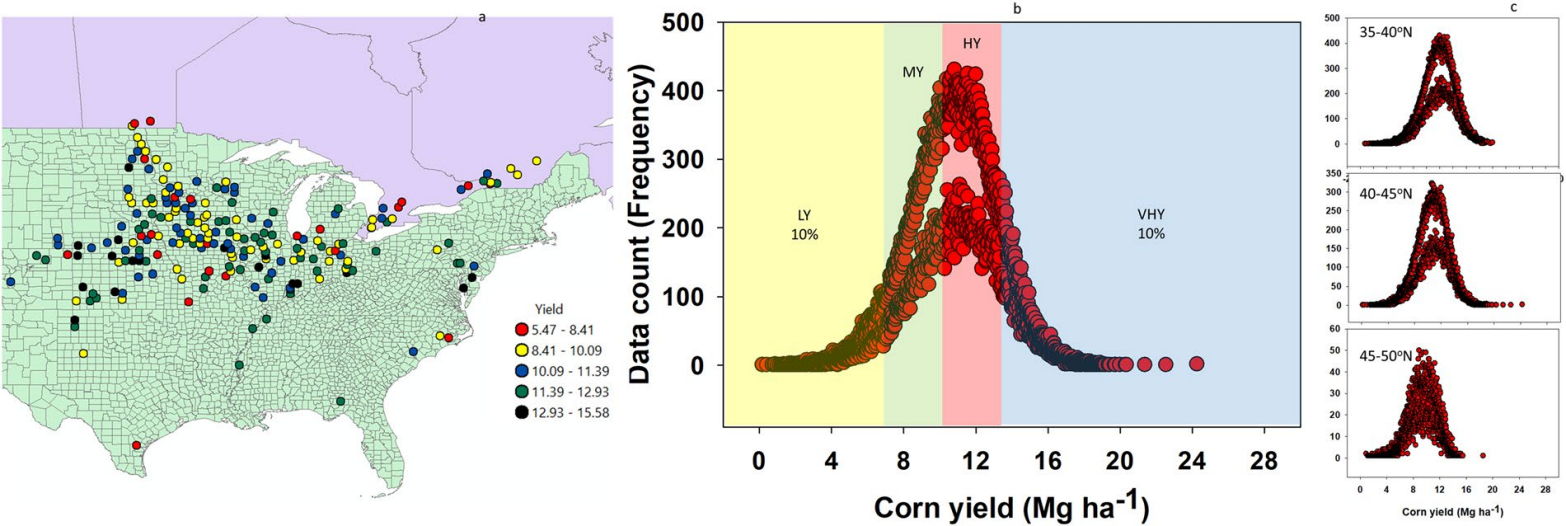
Location average yield classified in five groups (**a**), over all data distribution and yield environments (**b**), and yield distribution by three major latitude groups (**c**) for the maize hybrid seeding trial 1987–2016. Not all counties, states, provinces, or hybrids were present every year. LY, low yielding environment constituted the lower 10% of the data; MY, medium yielding environment; HY, high yielding environment; and VHY, very high yielding environment, which constituted the top 10% of the data.

This study is based on meta-data (187,662 data points) from 30 years of research on various plant density levels for maize hybrids released from the 1987–2016 period (Fig. 1). The average maize yield for the study period for each location ranged from 5.5 Mg ha^−1^ to 15.6 Mg ha^−1^ (Fig. 1a). When the entire data distribution was plotted, maize yield was approximately normally distributed with minimum yield of 0.2 Mg ha^−1^ to maximum yield of 24.3 Mg ha^−1^ (Fig. 1b). For the latitude groups, minimum and maximum yields ranged from 0.7–20 Mg ha^−1^ for the 35–40°N, 0.2–24.3 Mg ha^−1^ for the 40–45°N, and 0.9–18.5 Mg ha^−1^ for the 45–50°N (Fig. 1c).

**Figure 2:**
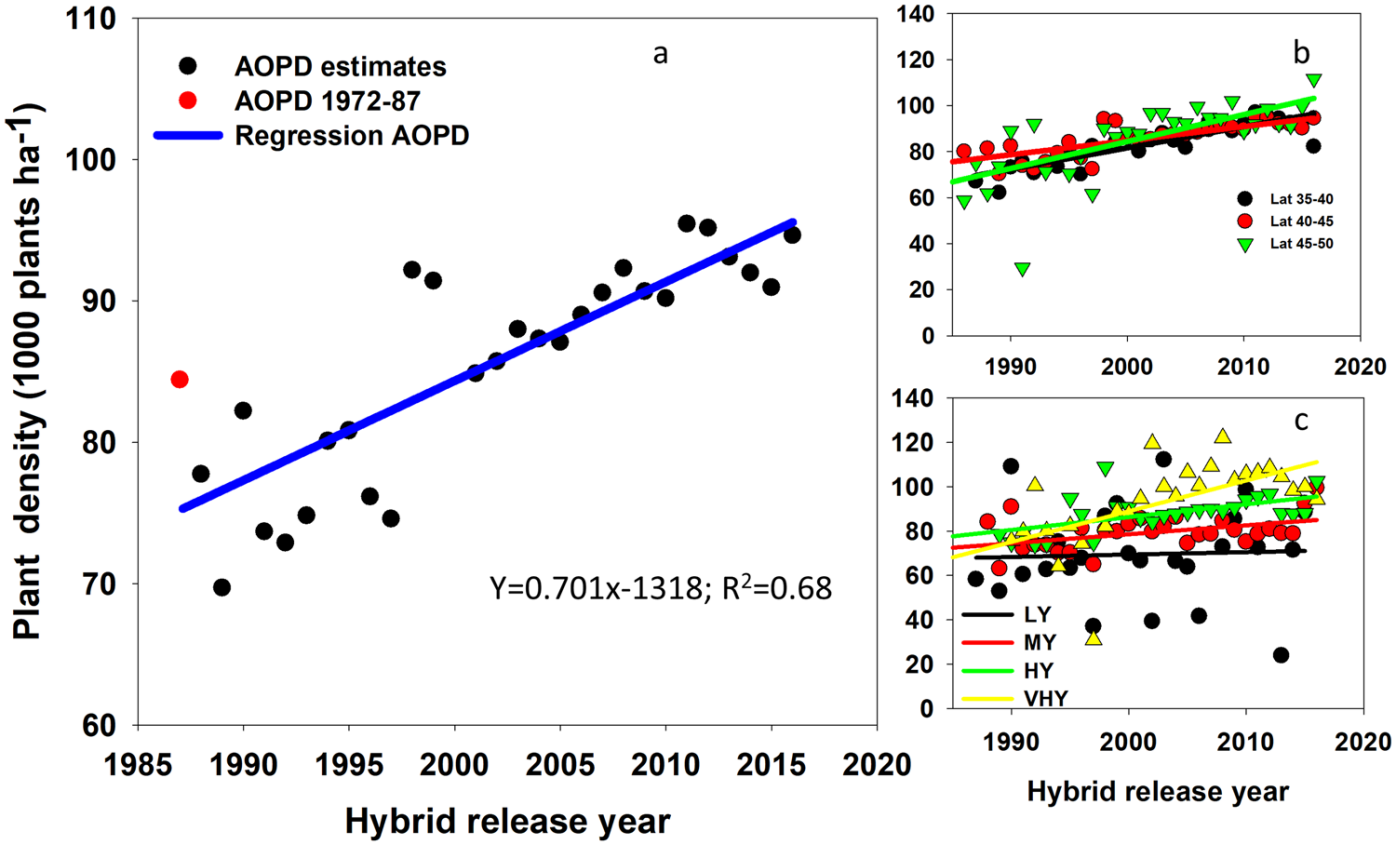
Changes in average agronomic optimum plant density (AOPD) over hybrid release year (panel a) for the entire North America, (panel b) by latitude group, and (**c**) by yield environment (LY, low-yielding; MY, medium-yielding; HY, high-yielding; VHY, very-high yielding environments). Red dot in panel a represent AOPD averaged for the hybrid release years 1972–87.

Agronomic optimum plant density increased at an average rate of 700 plants ha^−1^ yr^−1^ for the 1987 to 2016 period (Fig. 2a; Table 1). Estimated AOPD presented a year-by-year variability perhaps due to environmental conditions, i.e., as indicated by Assefa *et al*.^24^ the period between 1987 through 2016 included few years with wide ranged drought and other years with more favorable climate. The increase in average AOPD was a result of an increase in AOPD by 923, 611, and 1170 plants ha^−1^ yr^−1^ in 35°–40°, 40°–45°, and 45°–50° N latitude groups, respectively (Fig. 2b, Table 1). For yield environments, average increase in AOPD is primarily due to significant increases in both HY and VHY environments, 1386 and 611 plants ha^−1^ yr^−1^, respectively (Fig. 2c, Table 1). The AOPD did not significantly change for the LY environment, and only a moderate increase of 400 plants ha^−1^ yr^−1^ was documented for the MY environment.

**Table 1:** Simple linear regression equation of AOPD and yield at AOPD (Y) over hybrid release years (X) fitted at different hierarchical steps of modeling, coefficient of determination (R^2^), and standard error (SE) of the slope of the models.

No.	Related figure	Equation	R^2†^	SE of Slope
**Trend in AOPD**				
Y (1000*plants ha ^**−1**^ **); X (years)**				
1	Fig. 2a	Y_overall_ = 0.701 × −1318	0.68	0.06
2	Fig. 2b	Y_35–40_ = 0.923 × −1765	0.69	0.11
3		Y_40–45_ = 0.611 × −1138	0.60	0.09
4		Y_45–50_ = 1.170 × −2256	0.47	0.47
5	Fig. 2c	Y_VHY_ = 1.386 × −2683	0.35	0.26
6		Y_HY_ = 0.580 × −1073	0.33	0.11
7		Y_MY_ = 0.404 × −730	0.20	0.10
8		Y_LY_ = 0.110 × −150	0.001	0.49
**Yield trend at AOPD**				
**Y (Mg ha** ^**−1**^ **); X (years)**				
5	Fig. 3a	Y_overall_ = 0.149 × −285.6	0.91	0.01
5	Fig. 5a	Y_VHY_ = 0.028 × −41.9	0.56	0.003
6		Y_HY_ = 0.038 × −63.9	0.87	0.002
7		Y_MY_ = 0.027 × −45.1	0.77	0.002
8		Y_LY_ = 0.004 × −3.57	0.02	0.006

^†^The R^2^ values in this case indicated the relationship between mean yield, which is adjusted for variability by location or any other treatment, over the years.

### Yield trend at AOPD for the North America

**Figure 3:**
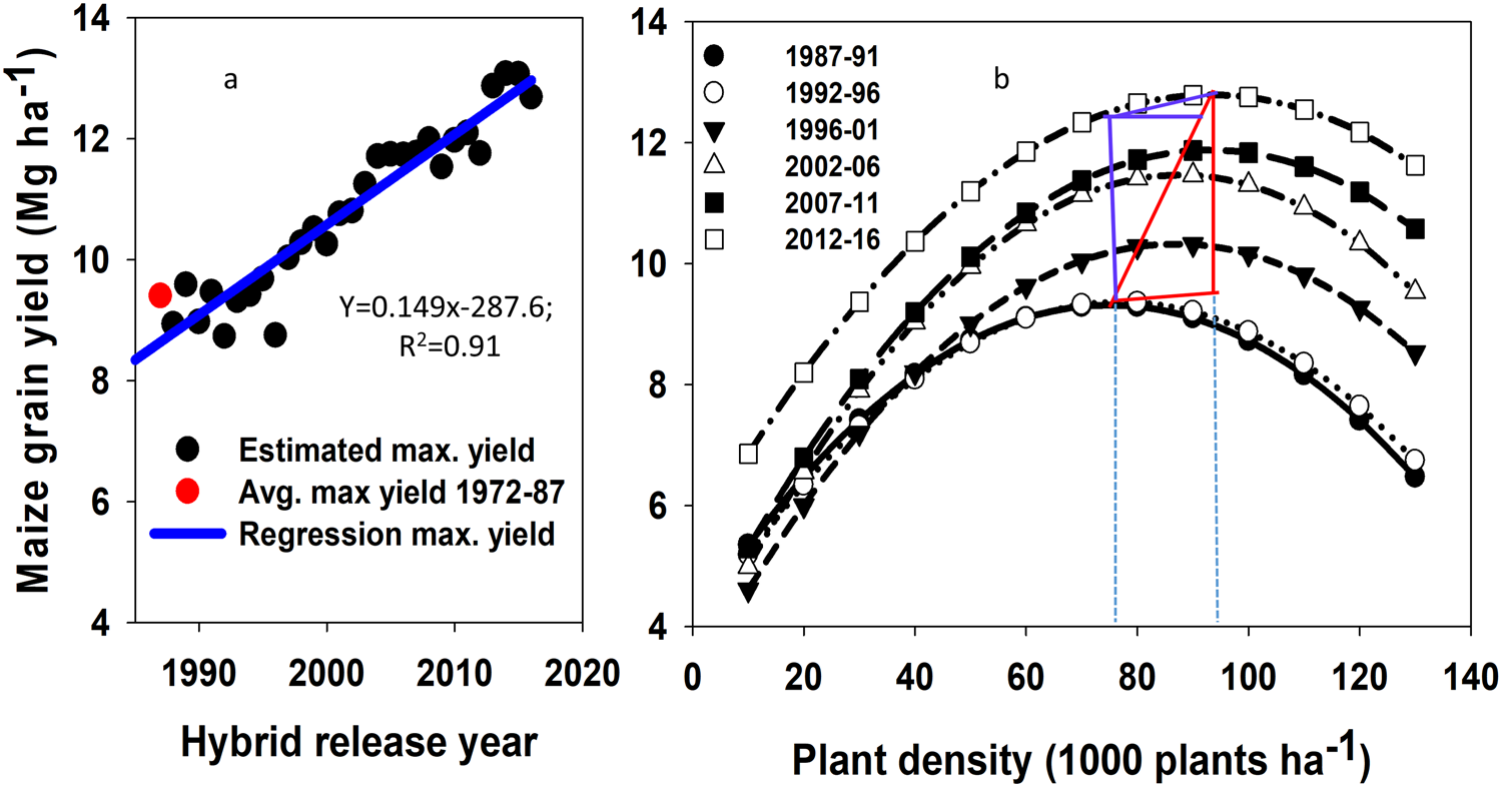
Trend in maximum maize grain yield at the agronomic optimum plant density (AOPD) over hybrid release year in North America (panel a) and models for the relationship between plant density and yield from 1987 through 2016 averaged over five-year periods. Red dot in panel a represent yield at the AOPD (average predicted maximum yield) for the hybrid release years 1972–87. In Panel b, red triangle represents the change in planting density in horizontal side, change in yield in the vertical side of the triangle, and the slope (change in yield over change in planting density) by the hypotenuse of the triangle. Vertical dashed (dotted) lines in panel b indicate the positions of the AOPD at each lustrum. Equations for panels a and b are included in Tables 1 and 2, respectively.

Yield at the AOPD (maximum yield) also increased significantly at the rate of 149 kg ha^−1^ yr^−1^ (Fig. 3a, Table 1); this yield gain rate is similar to the previously reported by Assefa *et al*.^24^. The AOPD ranged from 75 thousand plants ha^−1^ for the initial lustrum (1987–1991) and 93 thousand plants ha^−1^ for the final period (2012–2016), with yields moving from 9.3 to 12.7 Mg ha^−1^, respectively (Table 2, Fig. 3b). The quadratic curve (Fig. 3b) and the confidence interval for the AOPD point (Table 2) indicated that the AOPD range widened as it increased over the years. Not only did the AOPD increase over time it also broadened, i.e., the size of the plateau at the top of the quadratic curve widened presenting highest yields across a wider range of planting densities over the years. The 95% CI broadened from 8–9 thousand plants ha^−1^ between lower and upper limit during the 1987–1996 period to 10–12 thousand plants ha^−1^ for years 2007–2016 (Table 2). The model also suggested that yield increased over time across all plant densities but more significant changes occurred at greater plant density levels (Fig. 3b).

**Table 2:** Quadratic equations that best fit the yield-density relations by five-year periods (Fig. 3b) coefficient of determination (R^2^), and mean square error (MSE), agronomic optimum plant density (AOPD), 95% confidence interval (CI) of the AOPD, yield at the AOPD and at the upper and lower limits of AOPD.

NO.	Equation	R^2†^	Plant density (X)				Yield (Y) at		
	By lustrum		MSE = σ^2^	AOPD	95% CI		AOPD	95% CI of AOPD	
					lower	upper		lower	upper
				….(1000 plants) ha^−1^….			…………..Mg ha^−1^………….		
1	Y_1987–91_ = 4.03 + 0.14X − 0.0009X^2^	0.48	12.9	75.0	70.9	79.0	9.32	9.30	9.30
2	Y_1992–96_ = 3.84 + 0.14X − 0.0009X^2^	0.45	12.8	77.0	72.9	81.0	9.37	9.36	9.37
3	Y_1997–01_ = 3.02 + 0.16X − 0.0010X^2^	0.58	19.6	86.9	81.9	91.9	10.33	10.28	10.28
4	Y_2002–06_ = 3.20 + 0.19X − 0.0011X^2^	0.98	25.2	87.6	81.9	93.3	11.47	11.42	11.41
5	Y_2007–11_ = 3.62 + 0.18X − 0.0010X^2^	0.41	26.7	93.0	87.2	98.9	11.88	11.88	11.88
6	Y_2012–16_ = 5.34 + 0.16X − 0.0009X^2^	0.53	21.8	93.1	87.9	98.4	12.79	12.77	12.77

^†^The R^2^ values in this case indicated the strength of the relationship between mean yield-density in actual data over predicted by the quadratic model.

**Figure 4:**
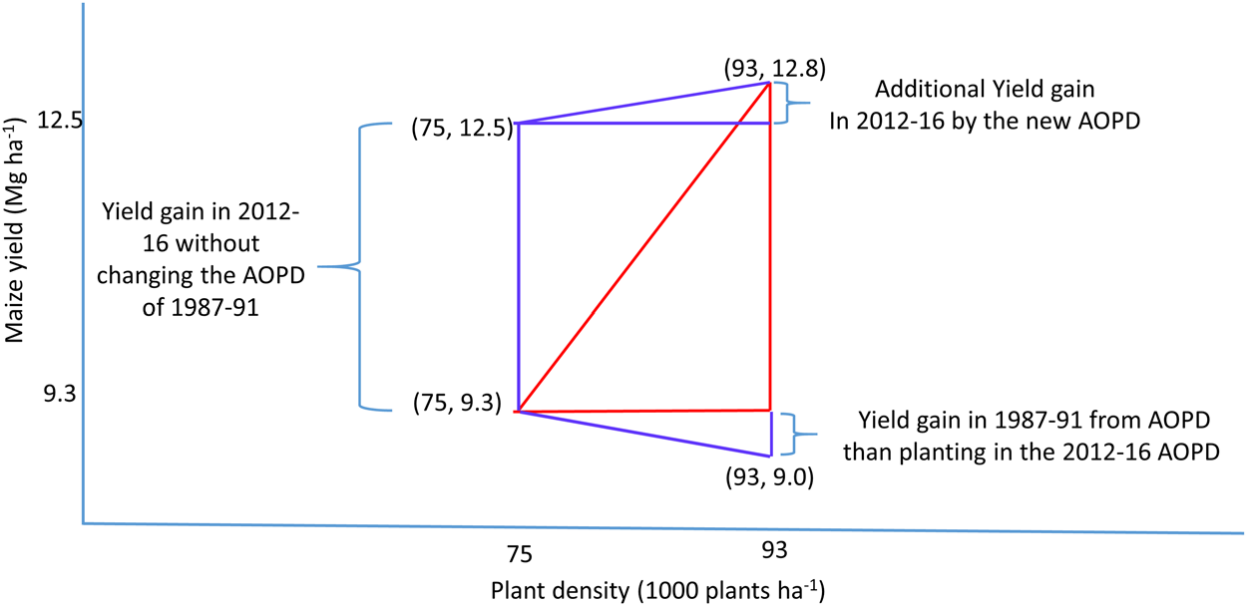
Maize maximum yield and AOPD averages for the 1987–1991 and 2012–2016 lustrums based on results in Fig. 3. The yield change without plant density change is the difference in yield of maize in 2012–2016 compared to 1987–1991 at the AOPD of 1987–1991. Yield change due to AOPD is the difference in yield gain at AOPD in 2012–2016 compared to yield for the same lustrum but at AOPD of 1987–1991. Additional yield gain from change in PD is the difference in yield of maize in 2012–2016 compared to 1987–1991 at the AOPD of 2012–2016.

From the total yield gain in the last six lustrums (1987–91 through 2012–16), two approaches were used (as indicated in methods section of this paper) to calculate the contribution of plant density to yield gain (Fig. 4). Using the first approach, yield gain was obtained of about 600 kg ha^−1^, which is about 17% of the total yield gain (3.5 Mg ha^−1^) at the AOPD (Fig. 4). In the second approach a yield gain was calculated of about 300 kg ha^−1^; which is 8.5% of the total yield gain between the two AOPD. (Fig. 4). Considering these two scenarios, the plant density contribution to maize yield in the last three decades is estimated to range from 8.5% to 17% (Fig. 4).

### Yield trend at AOPD by yield environments

**Figure 5:**
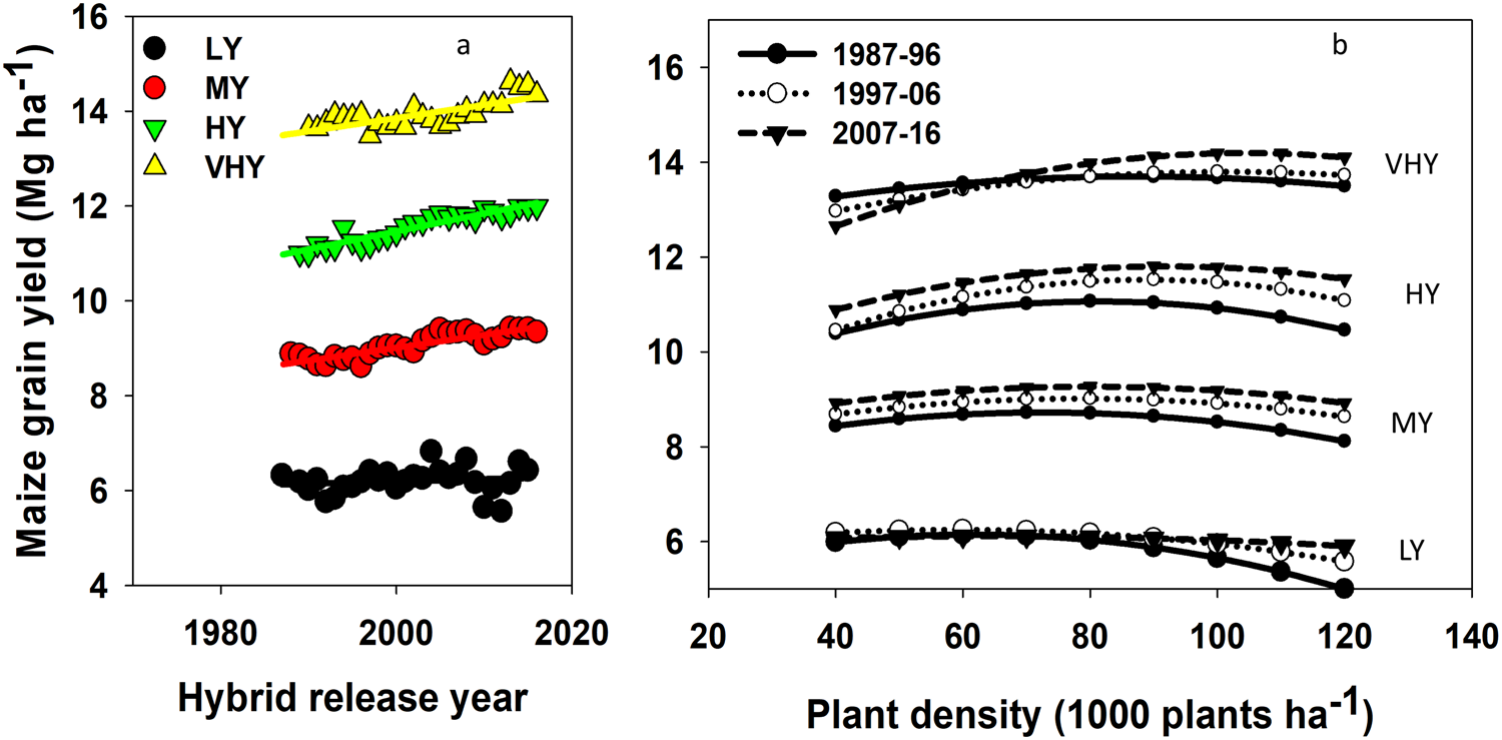
Changes in maximum maize grain yield at the agronomic optimum plant density (AOPD) over hybrid release year in North America (panel a) and relationship between yield and plant density for the recent three decades (panel b) all relative to yield environment (LY, low-; MY, medium-; HY, high-; and VHY, very high-yielding environments).

Yield gain rate at AOPD increased proportionally more as the yield environment improved from MY-to-VHY environments, from 28 to 38 kg ha^−1^ yr^−1^ (Fig. 5a, Table 1). These yield gain rates are significantly lower than the average yield gain rate previously reported of 149 kg ha^−1^ yr^−1^ for the North America region. The difference in those yield gain rates is likely because average regional rates of yield increase could be inflated by a significant yield increase in a single or few high yield environments and by an increase in the frequency of high yield environments^24^. The LY environments did not show a significant change in yield rate at the AOPD over time (Fig. 5a).

Yield changed across all plant densities in MY, HY, and VHY environments (Fig. 5b). For the MY environment, yield increase from the earliest- (1987–96) towards the latest- (2007–2016) decade seemed to be uniform across all plant density levels. For the HY and VHY environments, yield gain of modern relative to older maize hybrids is significant around the AOPD but is narrower at plant densities below the AOPD level. The yield-density relationship within 40–100 thousand plants ha^−1^ can be described as declining, constant, increasing to a plateau, and ever-increasing for LY, MY, HY, and VHY environments, respectively; in agreement to Assefa *et al*.^16^.

### Rate of change in yield to rate of change in AOPD

**Figure 6:**
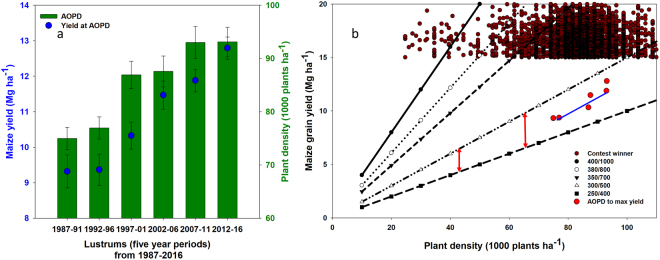
Changes in both optimal maize grain yield and AOPD over the last six lustrums (five-year periods) from 1987 through 2016 (panel a) and simulated relationship between plant density and yield at different seed weight (mg seed^−1^) to seed number (number of seed) combinations (panel b) in a high yielding environment with no resource limitation. A 400/1000 ratio indicates a hybrid that can produce 1000 seeds per plant with each seed weighing 400 mg. Also in the figure are example data points from the National Corn Growers Association Winner database (2011–2016) to demonstrate yields > 15 Mg ha^−1^ across different seeding rates and how our optimal planting density to yield data (red circles) fits into the simulation. Red two sided arrows in panel b indicate the change in yield gain at similar plant density between two hybrids differing only in seed weight and seed number. Error bars in (panel a) are standard errors.

The rate of change for yield gain outpaced the rate of change in plant density. For the lustrum-analysis (Fig. 6a), it can be noted that the change of yield at the AOPD significantly increased in each lustrum except for the first decade, while the rate of change of the AOPD significantly increased every decade from 1987 to 2016. For the 1987–91 to 2011–16 interval, AOPD increased by about 18 thousand plants ha^−1^ with yield increasing by 3.5 Mg ha^−1^, averaging 190 g yield per additional plant. The yield gain per plant was 138 g plant^−1^ for the years from 1987–96 to 1997–2006, but increased to 246 g plant^−1^ from 1997–2006 to 2007–16. The latter outcome indicates that average historical increase in plant density did not come with a decline in yield gain per plant.

In addition, it could be hypothesized that yield gain was not only due to increase AOPD, but it was also accompanied by a significant change in yield components, grain number and/or weight (Fig. 6b). To investigate this hypothesis, a simulation of yield from maize hybrids with five different grain number and grain weight combinations was pursued (Fig. 6b). The average grain number and grain weight trend over time for maize hybrids was mid-way between 250 mg grain^−1^ by 400 grains per ear to 300 mg grain^−1^ by 500 grains per ear or any other plausible variant of grain number to grain weight combination. Over time and as plant density increases, the data indicates (red dot trend in Fig. 6b) a slight shift from the side of 250 mg grain^−1^ towards 300 mg grain^−1^. Therefore, based on this simulation, yield gain did not only come from improvement of plant density but also from a *“per-se”* gain on yield components at the plant-level. Other reason that suggest factor other than planting density contribute to yield is the yield database gathered from the National Corn Growers Association (NCGA) contest winner (Fig. 6b), reporting yields above 15 Mg ha^−1^ across a wide plant density range.

## Discussion

### AOPD has increased over the years

**Figure 7:**
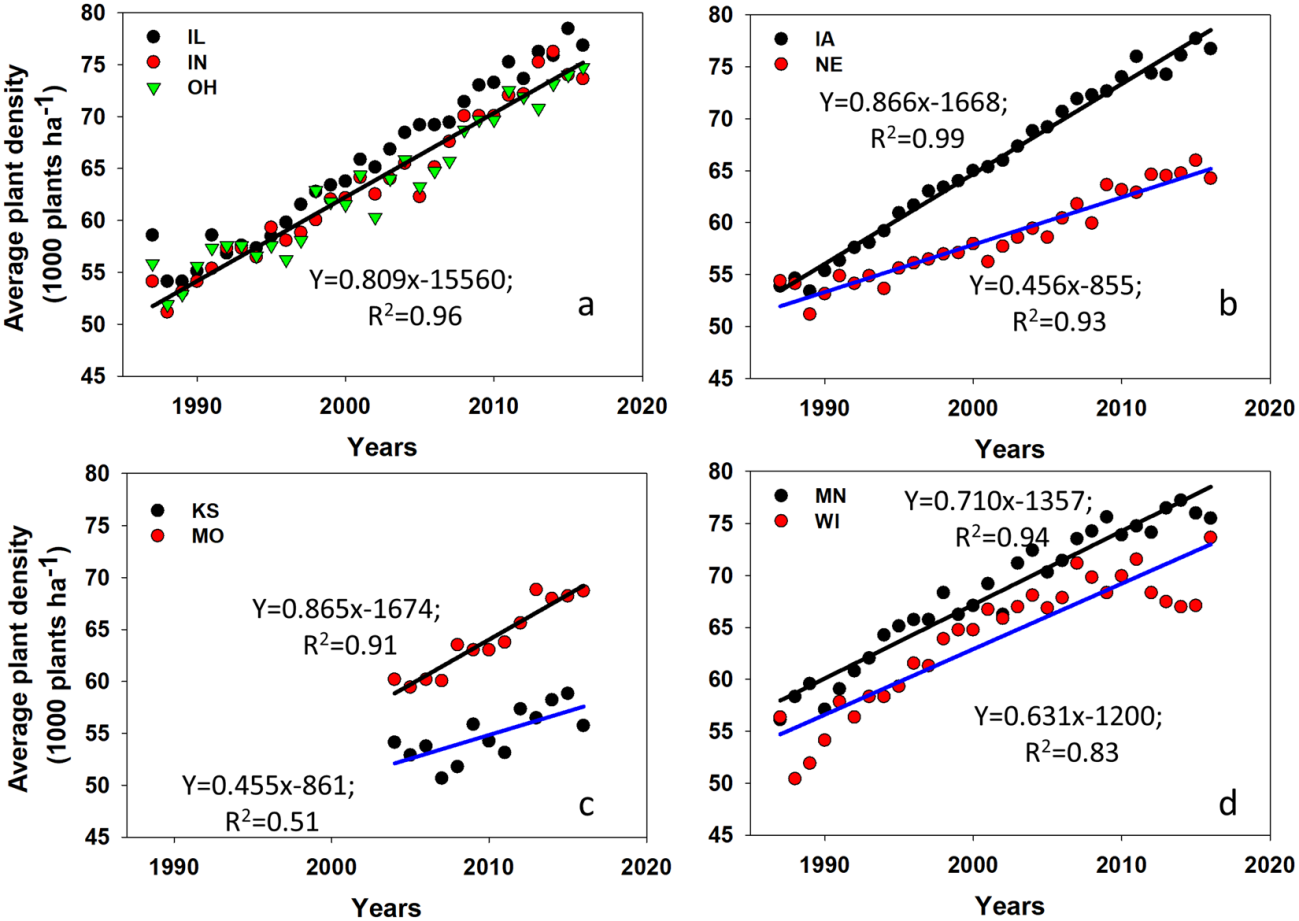
Trends in USDA reported average plant density for the years from 1987 through 2016 across nine states, which have significant maize production.

The first new contribution of this paper is the increase in AOPD at a rate of about 700 plants ha^−1^ yr^−1^ from 1987 to 2016. Even though it was well documented that plant density was the main management practice driving yield changes^1,2,7,8^, the rate of plant density change at the AOPD was not directly quantified and documented for the last three decades. Well referenced existing reports^1,27^ were also based on studies conducted in three selected planting densities and fewer environments compared with wider planting densities and many more locations that our results are based on. The rate of yield gain at the AOPD documented from this study is similar to the trend calculated from USDA for the main maize producing states for the same historical period^25^ (Fig. 7). Plant density increase from 450–870 plants ha^−1^ yr^−1^ was calculated for the seven states (IA, IL, IN, WI, NE, OH, and MN), but the majority presented a plant density increase of about 630–870 plants ha^−1^ yr^−1^ (Fig. 7). These plant density changes reported from DuPont Pioneer and USDA data are similar to previously reported planting density increase by Ciampitti and Vyn^7^ for the years 1984–2001 period, even when 100 scientific articles with diverse plant densities were synthesized (but AOPD was not documented in these articles).

### Yield at the AOPD increased significantly

The second new contribution is that yield at AOPD increased at a greater rate than previously documented in the scientific literature. For the current synthesis-analysis, an average yield gain at the AOPD of 149 kg ha^−1^ yr^−1^ was greater than the 90–130 kg ha^−1^ yr^−1^ reported in the US for the 1930–2010 period^12,24,26–29^. To the extent of our knowledge, this is the first time that average yield at the AOPD is documented utilizing a large database at a regional-scale with field research conducted in multiple sites and years. In addition, current average yield gain at the AOPD is primarily driven, as recently documented by Assefa *et al*.^24^, by an increase in the frequency of HY data points but also accompanied by positive changes in yield in the MY, HY, and VHY environments. When yield is compared between similar yield environments across years, no significant yield gain at the AOPD was recorded at the LY environment. This result is in agreement to the average yield gain by environment recently documented by^24^, even when yield gain was not reported at the AOPD but across all plant density levels tested.

### Change in AOPD and other yield components contributed to yield increase

The third new contribution is quantifying the variation in yield gain accounted for by the historical changes in AOPD, with AOPD contributing to approximately 17% to the last three decades gain on yield. This synthesis paper documented a yield gain at any similar planting density over the study time; however, the yield gap between earlier (1987–1991) and recent (2011–2016) maize hybrids increased as planting density increases. This yield change across all plant densities is an indication that, over the years, not only plant density but also other yield components contribute a significant portion of yield gain. For example, an increasing trend in kernel weight is previously documented for maize^30,31^ mainly for well-watered conditions. Ciampitti and Vyn^13^ documented an improvement in response to N fertilization for modern maize hybrids, with larger yield gap between modern versus old materials as the fertilizer N rate (and attainable yields) increased.

Yield increases due to other yield components are magnified at higher plant densities when yield environments are improved. In the past, researchers comparing old versus new hybrids at various plant densities documented smaller yield differences at lowest plant densities but a significant yield enhancement at higher plant density levels in favor to modern materials^2,32^. The increase in the yield enhancement between the old and new hybrid at higher plant density is the result of cumulative yield effects of small differences in grain number and weight, and in some cases number of ears per plant. Egli^33^ reported, based on data collected over literature, that kernels per ear and ear per plant did not change for US and Canada hybrids in the hybrid era but increased for Argentinian hybrids. Same study indicated a positive trend for kernel weight for US, Canada and Argentinian hybrids. Luetchensa and Lorenz^34^ recently reported an increase in leaf chlorophyll from old to newer hybrids, i.e., a per-plant morphological or physiological change, which could justify a per-plant yield change.

From a management standpoint, increases in AOPD and yield were feasible since maize producers specialized, i.e., moved from low to better yielding fields, and intensified production, i.e., adopted best management practices (BMPs) such as better hybrids, use of irrigation, improved nutrient fertilization, weed control, and other options in favorable environments^35^. These factors resulted in an increase on the proportion of HY and VHY environments and a decrease of LY environments. The relationship between plant-density and yield changed from negative in LY to positive in VHY environment^10^ (Fig. 5). Averbeke and Marais^21^ and Friedman^36^ reported an increase in critical plant density as water supply condition improves, decreasing plant density carrying capacity as the water deficit for the environment increases. It is also reported that, unlike the other management factors (transgenic insect resistance, fungicide containing strobilurin, P–S–Zn fertility, and N fertility) that improve yield independent of one another, plant density increased yield only when all factors were applied at a supplemental level^20^.

### Planting density and carrying capacity of corn environment

**Figure 8:**
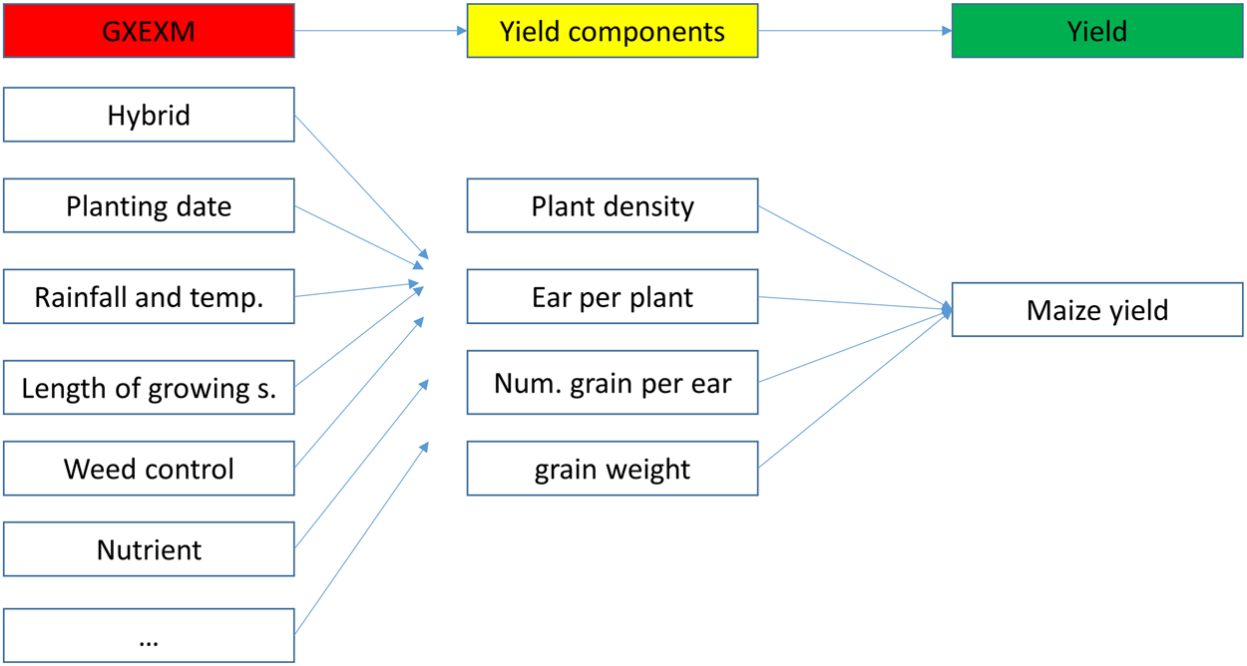
Theoretical framework on the effects of genotype (G) by environment (E) by management practices (M) effects on yield and its components, plant density, ear per plant, number of grains per ear, and grain weight. Plant density is a foundational intermediate factor that varies between- and is influenced by- G × E × M and thus affect yield (productivity). The G × E × M determines the yield environment.

A theoretical framework is presented suggesting that plant density is a foundational intermediary factor that varies with the G × E × M and productivity (Fig. 8). A change in plant density without improving productivity or yield environment, negatively impacts yield. Excessive plant density, defined as the plant density above carrying capacity of the environment decreases grain number, weight, and increases plant barrenness^37–40^. An improvement in yield environment via changes in G × E × M, increases the carrying capacity of the environment, consequently, resulting in an improvement of overall maize productivity. Therefore, plant density is reflecting the “carrying capacity” of any maize growing environment. Future analysis of the contribution of different factors to yield should consider the yield to plant density foundational relationship as more than just a standalone factor affecting maize yields. Research on modern maize hybrids should characterize their potential number of ears per plant, grain number per ear, and potential weight relative to their best-fitted environment. This provides a benchmark phenotypic evaluation of modern maize hybrids for factors contributing to yield improvement and can be used to evaluate progress at any point in time.

## Conclusion

The main outcomes from this synthesis were: (i) an increase in AOPD at a rate of 700 plant ha^−1^ yr^−1^, (ii) increase in AOPD across latitudes with rates of 923, 611, and 1170 plant ha^−1^ yr^−1^ for the 35° to 40°, 40° to 45°, and 45°-to-50° N latitude groups, respectively, (iii) increase in AOPD was 1386, 580 and 404 plants ha^−1^ yr^−1^ for VHY, HY, MY, respectively, but no significant change for the LY environment, (iv) 149 kg ha^−1^ yr^−1^ yield gain at the AOPD; (v) depending on the yield gain scenario, plant density contribution to maize yield gain ranged from 8.5% to 17%, (vi) an increase in yield across all plant densities, and (vii) yield improvement was partially explained by changes in the AOPD but improvements in yield components were identified as another source for yield gain. In summary, driven mainly by the increased proportion of favorable yielding environments, average AOPD and yield at AOPD increased over time. The findings synthesized in this review of a large meta-data suggest that a yield gain from modern maize hybrids was due to not only increased plant density tolerance, but also improvement in other yield components.

More research investment on understanding the changes related to the main yield components underpinning yield formation should be pursued to quantify potential sources to tap into for future maize yield improvement. Nonetheless, this work will require more integrated and multi-faceted inter-disciplinary research teams.

## Methods

Maize hybrid by seeding rate trials were conducted in 23 states in the US and 3 provinces in Canada by DuPont Pioneer® from 1987 through 2016. Within each state or province, field research was conducted in one or more counties. Not all counties, states, provinces or hybrids were present in the analysis every year. The experimental design for trials was a randomized complete block design (RCBD) with a split-plot arrangement. Plant density was the whole plot treatment and hybrids were in the subplot level. There were five target plant densities: 34,595; 44,475; 59,306; 74,132; and 88,958 plants ha^−1^ from 1987 to 1997; 44,475; 59,306; 74,132; 88,958 and 103,784 plants ha^−1^ from 2000-to-2010; and 44,475; 64,247; 84,016; 103,784 and 123,553 plants ha^−1^ from 2011-to-2016^10,18^. Hybrids included in the studies and analyses were 30 to 80 entries per year among important commercially available Pioneer products. The study was conducted in research sites and farmer fields with plot size of 3.05 m (4-rows) wide by 5.4 m long (0.76 m row spacing), and there were 2–5 replicates at each site. Plots were uniformly fertilized with all recommended nutrients for their respective region and related to their yield potential. Frequency distribution for all hybrids (Fig. 1a), data by plant density (Fig. 1b) and yield (Fig. 1c) relative to hybrid release year were explored.

Yield was recorded on the central two rows in each plot, grain moisture was measured, and yields adjusted to 155 g kg^−1^. Maize hybrid comparative relative maturity (CRM), maturity ratings were obtained from DuPont Pioneer® information^41^. These ratings are based on hybrid comparisons with maturity checks both at flowering and from grain harvest moisture levels of the hybrid.

Pre-analysis, data were grouped into three physical environment groups; 35°–40°, 40°–45°, and 45°–50° N latitude-based on median latitude of counties where trials were conducted. Here, physical environments are defined by latitude groups. Since, not all possible latitudes within the range have equal number of locations and also since not all locations were necessarily present every year, grouping within a latitude was preferred over treating latitude as a continuous variable. In this manner, grouping locations within a latitude results a representative average by buffering an experimental data in a latitude that might be collected in extreme (good or bad) weather conditions. Three physical environments were chosen because two (with 10°N range) will be small considering the majority of our locations are between 35°N and 50°N and four or more (with less than 5°N range) will result only small data sets in certain latitude groups.

Data were also grouped into four yield environments; low yielding, LY (<7 Mg ha^−1^), medium yielding, MY (7–10 Mg ha^−1^), high yielding, HY (10–13 Mg ha^−1^), and very high yielding, VHY (>13 Mg ha^−1^) based on maize yield obtained from each plot, following a similar procedure as previously detailed^16,18^. The LY environment represented the lowest 10% of the data and VHY environment represented the top 10% of the yield data both relative to all the data obtained across years and environments. From the maximum of the lowest 10% yield to the median yield represented the MY environment data and from the median to the lowest of the top 10% yield comprised the HY environment data (Fig. 1c). Based on this classification, assignment of site (plot) to a yield environment could change depending on the yield each year (i.e., one site could be LY one year and MY another year). The analysis was conducted using different approaches in terms of grouping data over time. A trend analysis was conducted using annual yield or plant density estimates or by grouping the data by decade or lustrum. Yearly estimates vary significantly depending on the condition of each year. Grouping of data by decade or lustrum as oppose to just by year results in steady model estimates and more representative of the average value of each environment. Data were grouped into six lustrums (five-year groups) based on hybrid release year: 1987–91, 1992–96, 1997–01, 2002–06, 2007–11, 2012–16; or into three decades: 1987–96, 1997–06, 2007–16.

Agronomic optimum plant density (AOPD) is herein termed as the plant density that results in maximum yield. The first step in our analysis was determining the annual AOPD based on experimental data points. To obtain the AOPD, the best-fitted quadratic model was selected by fitting the dependent variable yield against the independent variable plant density for each hybrid release year in SAS PROC MIXED procedure. After obtaining the best-fitted quadratic model per year; the AOPD, and yield at the AOPD in each year was determined using SAS response surface regression (PROC RSREG).

Second, a trend over-time for AOPD was determined by regressing the estimated AOPD over each year. Similar to global analysis, determination of AOPD and regression analysis were conducted by yield environment and by latitude. Also, a trend over-time for the yield at the AOPD was determined by regressing yield (maximum yield) at AOPD over hybrid release year.

Third, since annual AOPD fluctuated depending on the year, a comparison of AOPD and yield at the AOPD by lustrum was conducted. The best-fitted quadratic model for each lustrum was obtained by modeling the dependent variable yield against independent variables plant density for each lustrum in a mixed model. The AOPD value, and yield at the AOPD in each year, and mean square error (variance) associated to the plant density was determined using SAS response surface regression. Most statistical inferences (e.g., mean separation tests, confidence intervals) are applied for mean values of a data and statistical inferences for an optimal point (e.g., AOPD) or maximum point (e.g., maximum yield) are limited. Here, the mean square error (variance) associated to the plant density for each lustrum, obtained from PROC RSREG output, was utilized to calculate the 95% confidence interval (CI) - upper and lower limits (confidence interval) for the AOPD of each lustrum using Eqs [1] and [2].

1 $$s.e.\,(AOPD)=\sqrt{\frac{{\sigma }^{2}}{n}}$$

2 $$95 \% \,C.I.=AOPD\pm 1.96(s.e(AOPD))$$

where s.e.(AOPD) is standard error for the AOPD, σ2 is the variance associated to the plant density, 95% C.I. is 95% confidence interval for the estimated AOPD.

As a fourth step, from the total yield gain in the last six lustrums (1987–91 through 2012–16), two approaches were used to calculate the contribution of plant density to yield gain (Fig. 4). Using the first approach, yield gain due to changes in plant density was calculated as the difference between (1) yield gain in 2012–16 compared with 1987–91 both at the new AOPD (Eq. 3), minus 2) yield gain in 2012–16 compared with 1987–91 both at old AOPD (Eq. 4), divided by 3) total yield difference between 2012–16 and 1987–91 each at their respective AOPD (Eq. 5).

**Approach 1**

3 $$Yiel{d}_{2012-16,93}-Yiel{d}_{1987-91,93}=12.8\,Mg\,h{a}^{-1}-9.0\,Mg\,h{a}^{-1}=3.8\,Mg\,h{a}^{-1}$$

4 $$Yiel{d}_{2012-16,75}-Yiel{d}_{1987-91,75}=12.5\,Mg\,h{a}^{-1}-9.3\,Mg\,h{a}^{-1}=3.2\,Mg\,h{a}^{-1}$$

5 $$Yiel{d}_{2012-16,93}-Yiel{d}_{1987-91,75}=12.8\,Mg\,h{a}^{-1}-9.3\,Mg\,h{a}^{-1}=3.5\,Mg\,h{a}^{-1}$$

6 $$\frac{Equ.[3]-Equ.[4]}{Equ.[5]}\,\ast \,100=\frac{600\,kg\,h{a}^{-1}}{3.5\,kg\,h{a}^{-1}}=17 \% $$

where *Yield*_2012–16,93_ is average yield in the years 2012 through 2016 at the plant density of 93 thousand plants ha^−1^; *Yield*_1987–91,93_ is average yield in the years 1987 through 1991 at the plant density of 93 thousand plants ha^−1^; *Yield*_2012–16,75_ is average yield in the years 2012 through 2016 at the plant density of 75 thousand plants ha^−1^, and *Yield*_1987–91,75_ is average yield in the years 1987 through 1991 at the plant density of 75 thousand plants ha^−1^.

In the second approach yield gain due to change in plant density was calculated following three main steps: (1) total yield gain at AOPD for each lustrum minus (Eq. 5), minus 2) yield gain (1987–91 vs. 2012–16) at the 1987–91 AOPD, divided by 3) total yield gain at AOPD for each lustrum (Fig. 4).

**Approach 2**

7 $$Yiel{d}_{2012-16,75}-Yiel{d}_{1987-91,75}=12.5\,Mg\,h{a}^{-1}-9.3\,Mg\,h{a}^{-1}=3.2\,Mg\,h{a}^{-1}$$

8 $$Yiel{d}_{2012-16,93}-Yiel{d}_{1987-91,75}=12.8\,Mg\,h{a}^{-1}-9.3\,Mg\,h{a}^{-1}=3.5\,Mg\,h{a}^{-1}$$

9 $$\frac{Equ.[8]-Equ.[7]}{Equ.[8]}\,\ast \,100=\frac{300\,kg\,h{a}^{-1}}{3.5\,kg\,h{a}^{-1}}=8.5 \% $$

Fifth, trend in yield at the AOPD by yield environment was conducted by regressing the maximum yields obtained for each hybrid release year. In addition, the best-fitted quadratic model for yield environment by decade was obtained in a mixed model with the dependent variable yield against independent variables plant density. The best-fitted quadratic curve was then compared to determine how yield changed across plant density for each yield environment by decade combination.

## References

[1] Duvick DN (2005). The contribution of breeding to yield advances in maize (Zea mays L.). Adv. Agron..

[2] Tollenaar M, Wu J (1999). Yield improvement in temperate maize is attributable to greater stress tolerance. Crop Sci..

[3] Sangoi L, Gracietti MA, Rampazzo C, Bianchetti P (2002). Response of Brazilian maize hybrids from different ears to changes in plant density. Field Crops Res..

[4] Qian C (2016). Response of grain yield to plant density and nitrogen rate in spring maize hybrids released from 1970 to 2010 in Northeast China. Crop J..

[5] Derieux M (1987). Estimation du progres genetique realise chez les mais grain en France entre 1950 et 1985. Agronomie.

[6] Di Matteo J, Ferreyra J, Cerrudo A, Echarte L, Andrade F (2016). Yield potential and yield stability of Argentine maize hybrids over 45 years of breeding. Field Crops Res..

[7] Tollenaar M, Lee EA (2002). Yield potential, yield stability, and stress tolerance in maize. Field Crops Research.

[8] Tokatlidis IS, Koutroubas SD (2004). A review of maize hybrids’ dependence on high plant populations and its implications for crop yield stability. Field Crops Res..

[9] Gonzalez, V. H., Tollenaar, M., Bowman, A., Good, B. & Lee, E. A. Maize Yield Potential and Density Tolerance. Crop Sci. 58: (2018).

[10] Cardwell VB (1982). Fifty years of Minnesota corn production: Sources of yield increase. Agron. J..

[11] Shaw, L. H. & Durost, D. D. The effect of weather and technology on corn yields in the corn belt, 1929–1962. USDA, *Econ. Res. Ser. Agric. Econ. Rep*. 80 (1965).

[12] Assefa Y, Roozeboom KL, Staggenborg SA, Du J (2012). Dryland and irrigated corn yield with climate, management, and hybrid changes from 1939 through 2009. Agron J..

[13] Ciampitti IA, Vyn TJ (2012). Physiological perspective of changes over time in maize grain yield dependency on nitrogen uptake and associated nitrogen efficiencies: A review. Field Crops Res..

[14] Sangoi L (2001). Understanding plant density effects on maize growth and development: an important issue to maximize grain yield Ci. Rur..

[15] Tokatlidis IS (2013). Adapting maize crop to climate change. Agron Sustain Dev.

[16] Assefa Y (2016). Yield responses to planting density for US modern corn hybrids: a synthesis-analysis. Crop Sci..

[17] Poneleit CG, Egli DB (1979). Kernel growth rate and duration in maize as affected by plant density and genotype. Crop Sci..

[18] Daynard TB, Muldoon JF (1983). Plant-to-plant variability of maize plants grown at different densities. Can. J. Plant Sci..

[19] Lobell DB (2014). Greater sensitivity to drought accompanies. maize yield increase in the US Midwest. Science.

[20] Ruffo ML, Gentry LF, Henninger AS, Seebauer JR, Below FE (2015). Evaluating management factor contributions to reduce corn yield gaps. Agron. J..

[21] Averbeke WV, Marais JN (1992). Maize response to plant population and soil water supply: I. Yield of grain and total above-ground biomass. S. Afr. J. Plant Soil.

[22] Woli KP, Burras CL, Abendroth LJ, Elmore RW (2014). Optimizing corn seeding rates using a field’s corn suitability rating. Agron. J..

[23] Stanger TF, Lauer JG (2006). Optimum plant density of Bt and non-Bt corn in Wisconsin. Agron. J..

[24] Assefa Y (2017). A New Insight into Corn Yield-Trend from 1987 through 2016. Crop Sci..

[25] USDA-NASS. Quick stats. USDA-NASS, Washington, DC. https://quickstats.nass.usda.gov/Quick_Stats/ (accessed 8 Aug. 2017).

[26] Duvick, D. N. Genetic contributions to yield gains of U.S. hybrid maize, 1930 to 1980. In: W.R. Fehr, editor, Genetic contributions to yield gains of five major crop plants. CSSA Spec. Publ. 7. CSSA, Madison, WI. p. 15-47 (1984).

[27] Castleberry RM, Crum CW, Krull F (1984). Genetic grain yield improvement of U.S. maize cultivars under varying fertility and climatic environments. Crop Sci..

[28] Eghball B, Power JF (1995). Fractal description of temporal grain yield variability of 10 crops in the United States. Agron. J..

[29] Kucharik CJ, Ramankutty N (2005). Trends and variability in U.S. corn yields over the 20th Century. Earth Interact..

[30] Russell WA (1984). Agronomic performance of maize cultivars representing different eras of breeding. Maydica.

[31] Barker T (2005). Improving drought tolerance in maize. Plant Breed. Rev..

[32] Duvick DN, Cassman KG (1999). Post-green revolution trends in yield potential of temperate maize in the north-central United States. Crop Sci..

[33] Egli DB (2015). Is there a role for sink size in understanding maize-population-yield relationships?. Crop Sci..

[34] Luetchens, J. & Lorena, A. J. Changes in dynamic leaf traits in maize associated with year of hybrid release. Crop Sci. 58 (2018).

[35] Smith, S. *et al* Maize. In Yield Gains in Major U.S. Field Crops. CSSA Special Publication 33. ASA, CSSA, and SSSA, 5585 Guilford Rd., Madison, WI 53711-5801, USA. p. 125-171 (2014).

[36] Friedman SP (2016). Evaluating the Role of Water Availability in Determining the Yield–Plant Population Density Relationship. Soil Sci. Soc. Am. J..

[37] Alessi J, Power JF (1974). Effects of plant population, row spacing, and relative maturity on dryland corn in the Northern Plains: I. Corn forage and grain yield. Agron. J..

[38] Duncan WG (1984). 1984. A theory to explain the relationship between com population and grain yield. Crop Sci..

[39] Norwood CA (2001). Dryland corn in western Kansas: Effects of hybrid maturity, planting date, and plant population. Agron. J..

[40] Bruns HA, Abbas HK (2006). Planting date effects on Bt and non-Bt corn in the mid-south USA. Agron. J..

[41] DuPont Pioneer. Comparing maturity of Pioneer brand corn products (field facts). DuPont Pioneer, Johnston, IA. https://www.pioneer.com/home/site/us/agronomy/library/ compare-maturity-corn-products/ (accessed 13 May 2017; verified 20 June 2017).

